# Severe Vitamin D Deficiency as a Trigger for Metabolic Seizures in Infancy: A Case Series and a Comprehensive Review of the Literature

**DOI:** 10.3390/nu18152458

**Published:** 2026-07-27

**Authors:** Maria Oana Săsăran, Monica Grama, Cristina Roxana Mareș, Andreea Bianca Stoica, Rodica Demenciuc, Brîndușa Căpîlnă, Ancuța Lupu, Cristina Oana Mărginean

**Affiliations:** 1 Department of Pediatrics 3, “George Emil Palade” University of Medicine, Pharmacy, Science and Technology of Târgu Mures, Gheorghe Marinescu Street No 38, 540136 Targu Mures, Romania; oanam93@yahoo.com; 2 Doctoral School, “George Emil Palade” University of Medicine, Pharmacy, Science and Technology of Târgu Mures, Georghe Marinescu Street No 38, 540136 Targu Mures, Romania; 3 Department of Pediatrics 1, “George Emil Palade” University of Medicine, Pharmacy, Science and Technology of Târgu Mures, Gheorghe Marinescu Street No 38, 540136 Targu Mures, Romania; cristina_campean2005@yahoo.com (C.R.M.); andreeab.stoica@gmail.com (A.B.S.); marginean.oana@gmail.com (C.O.M.); 4 Pediatric Clinic I, County Emergency Hospital of Targu Mures, Georghe Marinescu Street No 50, 540136 Targu Mures, Romania; rodicatozlovanu@gmail.com (R.D.); brindusarc@yahoo.com (B.C.); 5 “Grigore T. Popa” University of Medicine and Pharmacy, 700115 Iași, Romania; ancuta.ignat1@umfiasi.ro

**Keywords:** hypocalcemia, seizures, infants, severe hypovitaminosis D

## Abstract

**Introduction:** Hypocalcemia represents a major cause of seizures in children in the absence of fever or infections. Hypovitaminosis D, usually associated with a lack of proper prophylactic regimens, can trigger those events. This case series aims to highlight two cases of hypocalcemic seizures in infants, attributed to severe vitamin D deficiency, with the aim of raising awareness regarding the importance of vitamin D prophylaxis. A narrative review of the literature is also provided, which highlights similar reported cases. **Methods:** We hereby report two cases of male infants (aged 4 months and 5 months) who presented to the emergency department of a tertiary pediatric center with seizures in the absence of fever or infections. Diagnostic work-ups revealed low levels of total serum calcium and ionic calcium deficiency. The cause of the hypocalcemia turned out to be severe vitamin D deficiency in both cases, caused by complete absence of vitamin D supplements since birth and during maternal pregnancy. In both cases, combined oral calcium and vitamin D supplementation led to complete resolution of symptoms and restoration of normal calcium levels. A comprehensive review of the literature is also provided, which focuses on pediatric studies and case reports that analyzed the prevalence, particularities, and outcomes of hypocalcemic seizures related to vitamin D deficiency. Articles including subjects with congenital or endocrine disorders that could have caused hypocalcemia were ruled out. **Results:** After accessing the full-length form of each article and applying the inclusion and exclusion criteria, 27 articles were included, namely 14 studies and 13 case reports. Hypocalcemic seizures caused by vitamin D deficiency are more common in infants, are usually linked to maternal hypovitaminosis D, and have a higher prevalence in developing countries. Most of the case reports depicting hypocalcemic seizures were distinguished through very low vitamin D levels. **Conclusions:** These case series emphasize the importance of vitamin D supplementation for the prevention of hypocalcemia, which constitutes a major metabolic cause of seizures in infancy. Nevertheless, maternal vitamin D supplementation during pregnancy is the only factor that can ensure the presence of satisfactory deposits in the newborn and prevent hypovitaminosis D-related complications.

## 1. Introduction

Seizures are the most common pediatric neurological disorder, which makes up approximately 1% of the presentations in pediatric emergency departments [1,2]. Structural etiologies represent the most common cause of non-infectious triggered seizures, especially in infants, and neuroimaging is often required for the identification/exclusion of cerebral anomalies [3]. In every child presenting with seizures in the absence of fever, a full laboratory work-up is required, which includes a complete blood count, blood urea nitrogen, creatinine, serum electrolyte levels, calcium, magnesium, and glucose levels [4].

Hypocalcemia represents a major cause of afebrile seizures in children, especially in developing countries [5,6]. Chronic hypocalcemia is often asymptomatic, whereas acute hypocalcemia can also lead to a variety of symptoms, including tetany, hyperreflexia, muscle spasms, or cardiac arrhythmias [7]. In neonates, symptomatic hypocalcemia can have both syndromic and non-syndromic causes, whereas calcium levels in infancy are influenced by various factors, such as vitamin D levels, parathormone (PTH) levels, skeletal storage, and dietary intake [8]. Vitamin D deficiency causes rickets, and severe hypovitaminosis D is a particular etiology of hypocalcemic seizures in infancy, especially associated with depleted maternal vitamin D deposits [9,10,11]. Previous studies have shown that most of the infants with hypocalcemic seizures have low vitamin D levels, accompanied by an increase in alkaline phosphatase levels and secondary hyperparathyroidism [12,13]. Appropriate vitamin D prophylaxis is recommended for both mothers and newborns/infants, as breast milk or formula feeding does not cover the vitamin D daily intake requirements, and a direct relationship between maternal and neonatal infant levels has been previously documented [11].

Hypocalcemic seizures are usually refractory to standard anticonvulsive treatment, requiring intravenous calcium boluses and afterwards intravenous calcium supplementation [14,15]. Calcium gluconate 10% is the preferred replacement therapy, in doses of 200–800 mg/kg/day [16]. Nevertheless, continuous enteral supplementation of calcium has also been regarded as a therapeutic success in neonates [17]. In case of concurrent hypovitaminosis D, daily regimens of oral vitamin D supplements at doses of 3.000–5.000 IU/day are recommended or, alternatively, “Stosstherapy” of a unique, high dose of 600.000 IU/day can be administered if the patient’s/maternal adherence to daily oral supplementation is put into question [18]. Moreover, to ensure therapeutic efficacy, magnesium and phosphate levels also have to be corrected when required [16].

The present case series aims to highlight two cases of hypocalcemic seizures in infancy, secondary to severe vitamin D deficiency, who did not benefit from any vitamin D supplement since birth, with a secondary goal to raise awareness regarding the importance of maternal and infant vitamin D supplementation. Furthermore, we aim to provide a comprehensive review of studies conducted on the same topic and similar cases reported in the literature.

## 2. Case Description

### 2.1. Case 1

In this case of a 4-month-old male patient, the paroxysmal events were described as one-minute generalized tonic seizures accompanied by fixed mydriatic gaze, profuse sialorrhea, perioral cyanosis, and atypical sucking automatisms, followed by spontaneous resolution. Following this event, the patient was admitted into a regional pediatric service, where three similar paroxysmal events occurred, for which the patient was administered three sequential doses of intra-rectal diazepam (2.5 mg per dose) for seizure management. Subsequently, the infant was transferred to a tertiary pediatric center for specialized diagnostic evaluation. Upon admission, clinical findings revealed a normally developed infant, slightly altered general state, and transient bradycardia (average HR 90 bpm), normalized after the administration of a single dose of intravenous atropine (0.1 mg iv). Anamnestic questioning revealed that this was the mother’s fourth child, and during the pregnancy she had not received any oral vitamin supplements, nor did the child following his birth. Laboratory findings showed a significantly low serum ionic calcium level (0.69–0.82 mmol/L) documented on serially performed ASTRUPs, in accordance with equally low total serum calcium levels (5.2 mg/dL). The rest of the blood work demonstrated hypochromic microcytic anemia and secondary hyperparathyroidism. The electrocardiogram (ECG) showed a prolonged QTc interval, primarily due to ST-segment lengthening. Aside from the hypocalcemia, the electrolyte panel showed no abnormalities. Serum 25-hydroxyvitamin D levels were profoundly low, confirming severe deficiency. Infectious etiologies were excluded via a TORCH panel, which indicated past exposure to cytomegalovirus (CMV), Toxoplasma gondii, and Epstein–Barr virus (EBV). Urinary calcium excretion (24 h urine calcium test) was performed to assess for potential metabolic losses, with a negative result. During hospitalization, the patient experienced multiple additional paroxysmal episodes; however, these remained self-limited and lacked severe clinical manifestations. Furthermore, a non-contrast cranial computed tomography (CT) scan was conducted, which showed no evidence of acute intracranial lesions or structural abnormalities. Therapeutic management involved intravenous infusions of 10% calcium gluconate (initially 200 mg/kg, subsequently titrated down to 100 mg/kg) diluted in a normal saline–glucose solution. Post-stabilization, the patient was transitioned to an oral maintenance regimen consisting of 345 mg of ionized calcium and 5000 IU of vitamin D3 daily. This therapeutic approach resulted in the complete resolution of clinical symptoms and the normalization of biochemical parameters, with no further paroxysmal events being observed.

At the one-month follow-up, the patient presented in good general condition, clinically stable and neurologically intact, with no recurrence of paroxysmal events. Laboratory investigations revealed elevated serum vitamin D levels (181.30 ng/mL) and a serum calcium level within the normal range (10.9 mg/dL), while all other biochemical parameters remained within reference ranges. Based on these findings, the treatment was modified to a maintenance dose of 1000 IU/day of vitamin D3, while calcium supplementation was withdrawn.

### 2.2. Case 2

In this second case, a 5-month-old male patient was brought by his caregivers to a tertiary pediatric center following the occurrence of an afebrile paroxysmal event at home. The episode lasted approximately one minute and was characterized by a fixed gaze, unresponsiveness to stimuli, perioral and nasal cyanosis, and fist clenching. Prior to this event, the patient had a three-day history of rhinorrhea, nasal obstruction, and a productive cough. Anamnestic questioning revealed that the infant was born from a normal physiological pregnancy with no significant past medical history. Upon admission, clinical findings revealed a slightly altered general state with drowsiness and mild skin pallor; otherwise, the clinical examination revealed no pathological findings.

A blood gas analysis (BGA) performed in the emergency department revealed a low serum ionic calcium level (0.69 mmol/L), normal inflammatory markers, and mild microcytic anemia. The patient was admitted for etiological investigation and the initiation of specialized therapeutic management. During the first two days of hospitalization, while receiving intravenous calcium replacement, the patient experienced multiple similar paroxysmal episodes; however, these remained self-limited, with a duration of less than 30 s, and did not require anticonvulsant medication. Calcium substitution was administered based on daily serial BGA evaluations. Over the first three days, serum ionic calcium levels documented on BGAs ranged between 0.68 and 0.86 mmol/L, despite initial therapeutic management with 10% calcium gluconate boluses via micro-infusions, followed by a dose of 200 mg/kg/day. No other electrolyte abnormalities were identified. By the fourth day, ionic calcium levels improved to 0.93–1.03 mmol/L, prompting a transition to an oral calcium maintenance regimen. During the intravenous calcium substitution, electrocardiogram (ECG) monitoring showed a QTc interval at the upper limit of normal. Echocardiography revealed a patent foramen ovale and a small patent ductus arteriosus with no hemodynamic impact.

Additional laboratory investigations confirmed severe vitamin D deficiency (levels below 3 ng/mL), low total serum calcium levels (5.2 mg/dL)—in accordance with the BGA values, and secondary hyperparathyroidism (parathormone (PTH) 238 pg/mL). The extremely low vitamin D levels were consistent with the complete absence of oral vitamin D supplements since birth. Blood work also demonstrated slightly elevated liver enzymes (aspartate aminotransferase (AST) 119 U/L, alanine aminotransferase (ALT) 65 U/L), mild microcytic anemia, and a mildly decreased thyroid-stimulating hormone (TSH), though peripheral thyroid hormones remained within normal limits. For the differential diagnosis of paroxysmal events, a TORCH panel was performed, which did not show any recent infections. Given that the patient experienced three febrile spikes during the first day of admission and rhinorrhea with nasal obstruction, rapid respiratory viral antigen testing (influenza A/B, severe acute respiratory syndrome coronavirus 2 (SARS-CoV-2), respiratory syncytial virus (RSV)) was performed, yielding negative results, and a chest radiograph revealed no structural abnormalities. Vitamin D replacement therapy was initiated at 2000 IU/day.

Considering the favorable clinical evolution, with the complete resolution of fever and paroxysmal events, the patient was discharged and continued an oral maintenance regimen of calcium and Vitamin D. At the one-month follow-up, laboratory investigations revealed a serum Vitamin D level of 34.40 ng/mL, as well as calcium (10.3 mg/dL), liver enzymes, and TSH levels within normal ranges. A vitamin D3 maintenance dose of 1000 IU/day was recommended, together with cessation of the calcium supplements. Table 1 illustrates the initial vitamin D and serum Ca levels, as well as the ones after the one-month follow-up, after Ca and vitamin D supplementation.

## 3. Review of the Literature

### 3.1. Methods

A systematic search of the Pubmed and Google Scholar databases was performed for articles focusing on the incidence, concurring factors, or peculiarities of hypocalcemic seizures at pediatric ages, due to underlying hypovitaminosis D. Search terms used were “hypocalcemia” AND “seizures” AND “vitamin D deficiency” AND “child”. Inclusion criteria consisted of studies and case reports that met the review’s objectives, published between 1 January 2000 and 31 May 2026, enrolling pediatric populations, and available in English in extensive formats. Case reports/series or studies reporting on adult populations or children with other underlying conditions (besides vitamin D deficiency), which could have triggered seizures or have been responsible for the hypocalcemia, were excluded. Articles in which the vitamin D status was unknown for the included subjects were also excluded. Furthermore, article types such as abstracts, letters to the editor, and communications were also left out of the present review.

Authors SMO, GM, and MCO were involved in the exclusion of duplicates. The title and abstract of each article were preliminarily checked in order to exclude articles that did not meet the review’s objectives. Afterwards, the same authors accessed the full length of the manuscript, where available, and mutually agreed upon inclusion or exclusion of each article, in line with the criteria listed above.

### 3.2. Results

**Figure 1:**
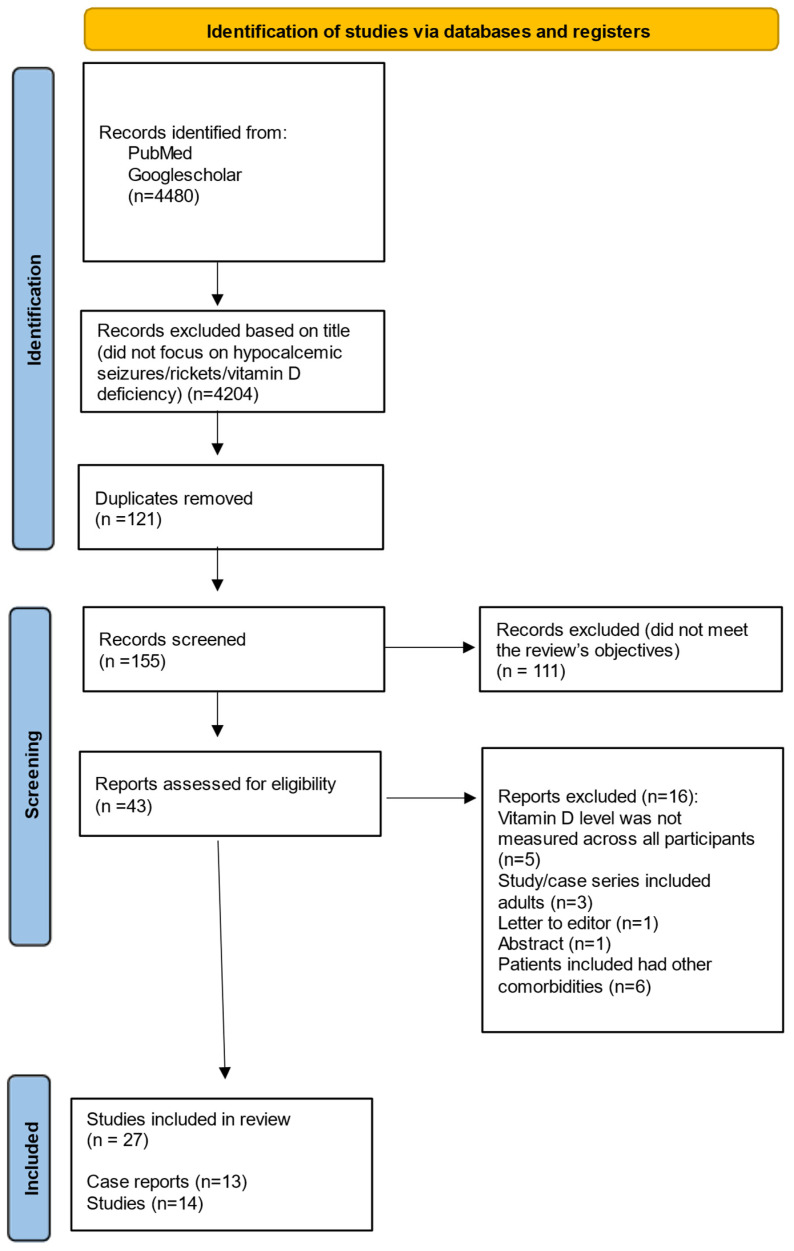
PRISMA flowchart for assessment of eligible studies.

After analyzing the abstracts and titles of the articles, 44 publications were initially included that corresponded to the review’s objectives. After accessing the full-length form of each article and applying the inclusion and exclusion criteria, 27 articles were included, namely 14 studies and 13 case reports. The article selection process has been schematically represented in Figure 1.

Hypocalcemia can manifest through life-threatening events, starting from early childhood. The absence of maternal vitamin D deposits can lead to multifocal, hypocalcemic seizures at neonatal ages, sometimes accompanied by refractory hypomagnesemia as well [19,20]. A distinction is made between neonatal early-onset hypocalcemia (manifesting in the first 3–4 days of life) and neonatal late-onset hypocalcemia (occurring in the 5th–10th day of life), also presenting through hypotonia, lethargy, pathological tremors, and myoclonic or focal seizures [21,22]. Early-onset hypocalcemia has been linked to DiGeorge syndrome, low birth weight, maternal gestational diabetes, or hyperparathyroidism, whereas late-onset hypocalcemia can be caused by maternal hypovitaminosis D, renal or parathyroid dysfunction [23,24,25]. One example of late-onset hypocalcemia was reported by Camadoo et al., who described a 7-day-old with a neonatal seizure linked to vitamin D deficiency. The neonate was born to a mother with negligible vitamin D deposits, who had not taken any supplements during pregnancy, but presented no signs of tetany [26]. In a study conducted on 10 newborns with hypocalcemia and generalized seizures, each newborn had severe vitamin D deficiency, correlated to similar maternal vitamin D levels, caused by an absolute lack of exogenous supplementation during pregnancy. Other symptoms of the clinical manifest hypocalcemia included craniotabes, which was present in 80% of the cases. No other clinical signs of rickets were identified [25]. Still, tetany attributed to vitamin D deficiency, subsequent hypocalcemia, and low ionized calcium has been described at ages as low as 50 days in an infant from Ethiopia. The main symptoms consisted of laryngospasm and breathing and feeding difficulties, manifesting through recurrent apneic spells. The mother did not take any vitamin D supplements during pregnancy and was also not exposed to sunlight, due to traditional religious dress code practices [27]. A case of status epilepticus, successfully controlled with intravenous midazolam infusion, was described in a 3-month-old, the ninth child, from a family with a positive history of epilepsy. The status epilepticus was caused by hypocalcemia in the context of severe hypovitaminosis D (4.3 ng/mL) [28].

In developing countries, severe pediatric hypocalcemia has been largely attributed to hypovitaminosis D and accompanied by notably low serum ionized calcium [29]. A Vietnamese study conducted on children with severe hypocalcemia concluded that very low levels of vitamin D and ionized serum calcium were predictors of seizures [29]. In higher-income countries, the incidence of hypocalcemic seizures related to vitamin D deficiency in children is relatively low, according to a surveillance study conducted in the United Kingdom and Northern Ireland. Still, male gender and a Black/South Asian heritage were particularly predictive of these conditions [30]. Another French study reported an incidence of around 21% for seizures in their study population with hypocalcemia [31]. For both studies, total serum calcium levels and vitamin D were even more decreased in the subgroups of children with seizures than in children without neurological manifestations [30,31].

A particularly vulnerable age group is represented by infants under the age of 6 months. In the study of Kamate et al., all infants with hypocalcemia and seizures were aged under 6 months and were exclusively breastfed. The study enrolled exclusively infants with seizures, in whom the prevalence of hypocalcemia was around 19% [32]. Vitamin D is almost untraceable in breast milk, and several cases of exclusively breastfed infants suffering from hypocalcemic seizures at very young ages have been reported, especially in the absence of exogenous supplementation [16,33]. Still, at neonatal ages, one Indian study showed that formula-fed neonates can develop hypocalcemic seizures if their mothers have not benefited from vitamin D supplements throughout the pregnancy [22]. Another study conducted in Nepal on infants and toddlers with seizures identified clinical signs of vitamin D deficiency in all subjects with hypocalcemia. These were breastfed or fed with both breast milk and formula [34]. Similar results were obtained by Balasubramian et al. within their study. All subjects with hypocalcemia were also vitamin D deficient, with all of them presenting secondary hyperparathyroidism [6]. The type of formula feeding might influence the development of vitamin D deficiency and related complications, even at infant ages. Rodd et al. presented a series of three cases of infants who developed hypocalcemia seizures due to vitamin D deficiency at around two months of age. Each infant had been fed soy-based formula and had received insufficient vitamin D supplements, considering their skin pigmentation [35]. Conjointly, these publications show that exogenous vitamin D supplementation in appropriate doses at very young ages is mandatory to ensure adequate calcium levels, independently of feeding patterns.

One case report from a Serbian hospital describes a 5-month-old male infant who presented with afebrile seizures, unresponsive to clonazepam. As in the two hereby reported cases, the patient presented severe hypocalcemia, low ionized calcium, extremely low serum vitamin D level (<3 ng/mL), secondary hyperparathyroidism, and high ALP levels [36]. The infant also presented signs of rickets, such as cupped deformation of the wrists, Harrison’s groove, and a rachitic rosary, was fed exclusively with cow milk starting from the age of two months, and had not received any vitamin D supplement since birth [36]. These two above-presented case series were also distinguished by an absolute lack of vitamin D supplementation since birth.

In another case of a 5-month-old infant from Greece, born at 36 gestational weeks and exclusively breastfed, afebrile seizures were related once again to hypocalcemia. This patient also presented extremely low levels of vitamin D (7.2 ng/mL), accompanied by hyperparathyroidism and an increase in ALP. Still, this patient did not present skeletal deformities [37]. At the same age, a Black 5-month-old infant, the third child, exclusively breastfed, presented with similar symptoms and similar modification of laboratory parameters: hypocalcemia, low ionized calcium, and almost non-existent vitamin D deposits (0.8 ng/mL). No clinical signs of rickets were visible, but wrist abnormalities were found on the X-rays. The mother had not benefited from any vitamin D supplement during pregnancy nor during breastfeeding [38]. The importance of vitamin D supplementation during pregnancy for the offspring’s vitamin D deposits had been previously highlighted by various studies [11,39,40]. A case–control study conducted in India showed that infants born to mothers with extremely low vitamin D levels, of under 10 ng/mL, pose the highest risk for hypocalcemic seizures [11]. Research performed on a cohort of Egyptian infants diagnosed with rickets proved that those with hypocalcemic seizures came from mothers with severe hypovitaminosis D [10]. On the same note, another study enrolling 60 infants with hypocalcemic seizures identified a vitamin D deficiency in each subject, which was linked to insufficient maternal deposits in most cases [13]. Furthermore, a previous study conducted in Romania demonstrated a clear, inverse relationship between maternal vitamin D levels and the number of pregnancies [40]. Hence, the higher the number of pregnancies, the higher the likelihood of having a newborn without vitamin D deposits, especially in the absence of maternal vitamin D supplement intake during pregnancy.

The largest set of data regarding infants with hypocalcemia and seizures comes from a study performed in Turkey. The study included 90 infants, with a mean age around 6 months, none of them with a previous history of vitamin D supplementation. More than 70% of the enrolled population were males, and almost all patients (more than 90%) presented secondary hyperparathyroidism and a significant increase in alkaline phosphatase levels. Only 37 patients presented vitamin D deficiency (defined as values < 20 ng/mL), but skeletal abnormalities compatible with rickets were found in 75% of the patients in radiological studies. Still, abnormalities in phosphorus levels were identified in less than 30% of patients, which is consistent with the two case reports presented by our team, in which hypophosphatemia was absent [12]. In contrast, in another study targeting an infant population from Bangladesh with hypocalcemic seizures, vitamin D deficiency was identified in all subjects, without exception [13]. An older study performed in Catalonia highlighted a higher incidence of rickets in immigrants of African origin and particularly described tetany and seizures as manifestations of vitamin D deficiency in infants under the age of 6 months, with a generalized pattern [41]. The neurological prognosis of infants admitted with hypocalcemia-induced seizures was analyzed after a time span of 6 months, within Turkish research enrolling 32 patients, predominantly males (over 70%). No disparities in the normal development of social and language skills were identified, but the acquisition of fine and gross motor skills experienced significant delays [42]. A summary of studies that analyzed the prevalence and outcomes of hypocalcemic seizures, as well as the impact of hypovitaminosis D on this metabolic imbalance, has been provided through Table 2.

Hypocalcemia seizures related to hypovitaminosis D have been reported in school-aged children and teenagers as well. A case series article showed that newly onset seizures can also take place at older ages, including during teenage and young adult years, also in relation to vitamin D deficiency-related hypocalcemia [43]. Tekin et al. described the case of a 6-year-old boy who presented with a 5 min, generalized seizure followed by spontaneous recovery. Hypocalcemia related to moderate vitamin D deficiency was identified as causing the seizure [44]. Schnadower et al. reported the case of a 17-year-old who presented with hypocalcemic seizures and bilateral femoral neck fractures in the absence of trauma. These events were attributed to primary vitamin D deficiency [45]. In both cases, the children had an unbalanced diet, which included regular fast food-based meals and insufficient dairy intake [44,45]. A diet based on multiple allergen exclusion can also lead to hypocalcemia and seizures through insufficient vitamin D intake at adolescent ages, according to a case report published by Hershberger et al. [46].

## 4. Discussions

Vitamin D maintains calcium homeostasis by stimulating its absorption within the gastrointestinal tract and its reabsorption within the distal renal tubules, together with parathyroid hormone. Calcium plays an important role in the release of neurotransmitters and in muscular contraction. Ion channel families that influence neuronal excitability are externally modulated by calcium through the calcium-sensing receptors (CaSR). These CaSR also send intracellular secondary signals through pathways such as cyclic adenosine monophosphate (cAMP). Calcium is therefore involved in the maintenance of the membrane resting potential and in the modulation of action potential frequency [47,48]. Animal model-based studies have shown that hypocalcemia enhances neuronal excitability by increasing the frequency of the action potential and by modifying the threshold potential to an almost equal value to that of the resting membrane potential [49,50].

This case series distinguished itself through the absence of clinical symptoms of rickets or tetany, as preceding or accompanying hypocalcemic seizures in infants with extremely low vitamin D levels. The favorable outcome after vitamin D and calcium supplementation further emphasizes that hypocalcemia was the only underlying cause of seizures. Moreover, these cases highlight the importance of vitamin D supplementation during both pregnancy and infancy for the prevention of hypocalcemia and neurologic-related complications. The appropriate prophylactic doses of vitamin D have been subject to much debate and varied greatly among case–control studies. The latest Clinical Practice Guidelines of the Endocrine Society recommend routine prophylaxis involving vitamin D supplements for the prevention of nutritional rickets and respiratory tract infections in children aged between 1 and 18 years. The doses asserted by the clinical trials reviewed ranged between 300 and 2000 IU, with an average of 1200 IU. No specific recommendations were issued by this guideline with respect to children younger than 1 year. Still, particular recommendations were issued during pregnancy, with empirical supplementation aiming to reduce the incidence of conditions such as pre-eclampsia, as well as premature birth and neonatal mortality. During pregnancy, daily doses of 600 IU to 5000 IU were provided in clinical randomized trials, with an average of 2500 IU/day [51].

In infants, older European recommendations advised a daily intake of 800–1000 IU/day [52,53]. However, given the possible risk of excessive intake, new World Health Organization (WHO) guidelines limit the recommended intake to 400–800 IU of vitamin D/day [54,55]. A recent systematic review focusing on infants indicated a usual dose of vitamin D of 400 IU/day for the general population, with higher doses, ranging between 600 and 1200 IU/day, mainly targeted at infants with a risk of deficiency, such as those with darker skin pigmentation or those who had limited sunlight exposure [56]. A Cochrane systematic review analyzed studies and clinical trials conducted on children younger than 5 years and analyzed the benefit of higher doses of vitamin D versus lower, usually used prophylactic doses for the prevention of respiratory tract infections. A higher vitamin D dosage did not decrease the number of healthcare visits related to respiratory tract infections but was also unlikely to cause hypercalcemia in either young children or pregnant women [57].

In this case series, the two infants were born to mothers who received no vitamin D supplement during pregnancy. Moreover, the Romanian ethnicity of the two infants placed them into a vulnerable population segment. These ethnicities are particularly prone to vitamin D deficiency and related complications, as brought to light by a previous study conducted in Romania, enrolling only the Romanian population. Within this study, enrolling mothers and their newborns, every subject presented vitamin D insufficiency or deficiency [58]. Thus, in these populations at risk, healthcare policies should aim to identify hypovitaminosis D and ensure adequate treatment to restore the corresponding deposits. The adequate vitamin D treatment dose still varies greatly, with recommendations of oral vitamin D supplements ranging between 1000 and 5000 International Units (IU)/day, given for 2–3 months, with later return to baseline prophylactic doses of 400–800 IU/day, as proposed by the American Academy of Pediatrics [59,60]. In this case series, the infants had two different attending physicians who prescribed different doses of vitamin D supplements. In the first case, receiving a vitamin D supplement totaling 5000 IU/day, very high levels were detected at the 1-month follow-up. The second case received a dose of only 2000 IU/day, which produced a milder increase in serum vitamin D, barely above the 30 ng/mL threshold. Hence, the recommended therapeutic doses should be adapted to the initial vitamin D levels.

A summary of the paraclinical data, nutrition, and vitamin D supplementation patterns of newborn and infant cases with hypocalcemic seizures is provided in Table 3. All cases presented good outcomes after calcium and vitamin D supplementation, with cessation of seizures. In most cases, vitamin D levels were extremely low, being almost untraceable in some instances [23,24,28,35,36,38]. In the above-presented case series, vitamin D levels could also not be quantified, and secondary hyperparathyroidism was also found, in line with other cases reported. With the exception of one case that received a low dose of exogenous vitamin D and another one with an appropriate prophylactic dose of daily vitamin D (400 IU/day), none of the infants from the publications summarized in Table 3 had received any vitamin D supplements, in similar fashion to our cases [24,35]. Still, the two cases of late-onset neonatal hypocalcemia were directly linked to maternal hypovitaminosis D, as vitamin D supplements are not recommended in the first week of life [19,26]. A common limitation of these case reports remains the lack of knowledge regarding maternal vitamin D status and the absence of data regarding vitamin D supplementation during pregnancy. In only two publications, the serum maternal level of vitamin D was quantified and reported [19,24]. The present case series also lacks information regarding maternal vitamin D levels, which were likely to be significantly low, considering the absence of maternal vitamin D supplements during pregnancy. Moreover, in spite of an important proven link between maternal and neonatal vitamin D levels, most of the studies conducted on infants with hypocalcemia and related metabolic seizures did not report maternal serum vitamin D levels, the amount of vitamin D supplementation, and did not analyze possible associations between maternal and neonatal vitamin D levels (Table 2). Future studies are warranted to deliver more information regarding the importance and appropriate dosage of vitamin D prophylaxis during pregnancy, for the prevention of metabolic-related complications in neonates and small infants.

## 5. Conclusions

Severe hypovitaminosis D can cause hypocalcemia-related seizures in infancy. A prompt recognition of metabolic causes of afebrile seizures is required in pediatric populations in order to ensure the correct and appropriate therapeutic intervention and to avoid adverse neurological outcomes. The two case reports and the literature data reviewed once again emphasize the importance of vitamin D supplementation during pregnancy and infancy for the prevention of hypocalcemia and its related life-threatening complications. Assessment of hypocalcemia-related risk factors includes a meticulous anamnesis regarding vitamin D supplementation and its subsequent dosage during pregnancy and after birth. Conjointly, the reviewed articles and case reports also suggest that in each child with hypocalcemic seizures, diagnostic workup needs to include at least magnesium, phosphorus, serum vitamin D testing, and parathormone level assessment for appropriate therapeutic management and identification of congenital or endocrine disorders.

## Figures and Tables

**Table 1:** Timeline of serum calcium and vitamin D levels: initial values and one-month follow-up values after vitamin D and Ca supplementation.

Cases	Initial Serum Ca Level (mg/dL)	Follow-Up Serum Ca Level (mg/dL)	Initial Serum 25-OH-Vitamin D Level (ng/mL)	Follow-Up 25-OH-Vitamin D Level (ng/mL)	Daily Vitamin D Supplement (IU)
Case 1	5.2	10.9	<3	181.3	5000
Case 2	5.2	10.3	<3	34.4	2000

**Table 2:** Vitamin D deficiency, hypocalcemia and hypocalcemic seizures in infants and children: summary of reviewed studies.

Author, Year, Country	Type of Study	Population Included	Main Outcomes
Balasubramanian et al., India, 2006 [6]	Observational prospective study	50 infants (2–6 months) with hypocalcemia	In 13 breastfed infants, seizures were secondary to VDD All infants had ↑ PTH, ↓ 25(OH)D All the mothers of the infants had ↓ 25 (OH)D Need to supplement Vitamin D in exclusively breastfed infants
Salama & El-Sakka, Egypt, 2010 [10]	Cohort study	32 breastfed infants with rickets + their mothers	9 (28%) infants had hypocalcemic seizures → their mothers had severe VDD (*p* = 0.005) 23 (72%) infants → vitamin D < 20 ng/mL
Mehrotra & Marwaha, India, 2010 [11]	Cross-sectional study	120 infants (aged 15 days–6 months) + their mothers divided into: ▪ 60 infants + their mothers with hypocalcemic seizures ▪ 60 healthy breastfed infants + their mothers	Hypovitaminosis D in 90% of study group vs. 41.7% of controls. Vitamin D deficiency (VDD) → major cause of hypocalcemic seizures
Cesur et al., Turkey 2013 [12]	Retrospective study	91 hypocalcemic infants (15 days–24 months) with rickets presenting as hypocalcemic convulsions	Infants had ↓ Ca, ↑ ALP, ↑ PTH; 25-OHD < 20 ng/mL None of the infants received prophylaxis
Biswas & Hasan, Bangladesh, 2021 [13]	Cross-sectional, hospital-based (35 months)	60 infants (age 3.6 months) with hypocalcemic seizures + their mothers	All infants had ↓ Ca, ↑ ALP, ↑ PTH 40% severely VDD, 60% deficient in vitamin D Positive correlation between infant and maternal 25(OH)D
Soliman et al., Qatar, 2013 [25]	Observational study	10 newborns (first 10 days of life) with symptomatic hypocalcemia 100 normal newborns 18 infants with rickets = controls	All newborns exclusively breastfed → with 25(OH)D < 10 ng/mL Generalized convulsions (10/10), craniotabes (8/10); low 25(OH)D (13.2 ng/mL) and maternal (8.1 ng/mL).
Nguyen et al., Vietnam, 2026 [29]	Retrospective cross-sectional study	246 children (0–18 years), of whom 24.8% had congenital heart diseases	79.2% with seizures 67.1% with VDD Children with VDD had ↓ 25(OH)D (18.4 mml/L), ↓ ionized Ca & ↑ PTH Severe hypocalcemia was associated with seizures
Basatemur & Sutcliffe, UK and Ireland, 2015 [30]	Prospective study (BPSU methodology)	91 children aged 0–15 years with hypo-calcaemic seizures due to VDD	Annual incidence of hypocalcaemic VDD seizures = 3.49 per million children aged 0–15 years (95% CI 2.81–4.26). Higher incidence in males, in infants vs. older children,
Flot et al., France, 2020 [31]	Retrospective study	38 children with rickets (age 23 months–5 years)	Hypocalcemic seizures 21%, Growth retardation 11%; 62% hypocalcemia. Children seizures → ↓ Ca and 25(OH)D
Kamate et al., India, 2018 [32]	Prospective observational study	78 normally developing children (1 month–2 years) with seizures	18 (23.1%) children with hypocalcemia 15 (19.2%) children secondary to hypovitaminosis D 41.67% of infants < 6-month exclusively breastfed infants had hypocalcemia (*p* = 0.001)
Qureshi et al., India, 2020 [22]	Prospective study	14 formula-fed neonates with late-onset neonatal hypocalcemia (aged 7.5 days)	All newborns had seizures due to late-onset hypocalcemia (Ca < 5 mg/dL), maternal value: 25(OH)D = 43 nmol/L Revisits VDD/high-phosphate formula as a cause of late-onset neonatal hypocalcemic seizures.
Ramesh Bhat et al., Nepal, 2025 [34]	Prospective observational study	58 children (1 month–2 years) with afebrile seizures and their mothers	6 (10.3%) had hypocalcemia with clinical VDD signs (2 severe VDD with ↑ ALP + PTH. Generalized-onset seizures predominated (62%)
Yeste & Carrascosa, Spain, 2003 [41]	Retrospective clinical survey (10 years)	62 infants and children (34 boys, 28 girls, age 9.9 months) with rickets	Most infants < 6 months → hypocalcemic tetany/seizures
Kaya Özcora et al., Turkey, 2022 [42]	Retrospective study (1-year follow-up)	32 patients (6 days–24 months) with hypocalcemic seizures due to VDD	Infants had ↓ Ca, ↑ ALP, ↑ PTH, 25(OH)D 9.6 ng/mL; maternal 25(OH)D = 7.16 ng/mL Improvement in gross and fine motor development at 6 months (*p* < 0.001) VDD/hypocalcemia can cause reversible psychomotor delay.

Legend: ↓—decrease; ↑—increase; ALP—alkaline phosphatase; PTH—parathormone; VDD—vitamin D deficiency.

**Table 3:** Case reports of newborns and infants with hypocalcemic seizures related to vitamin D deficiency: laboratory values and nutritional characteristics.

Publication Author, Year, Reference	Age	25-Hydroxy-Vitamin D Level (ng/mL)	Calcium (mg/dL)	Ionized Calcium (mg/dL)	ALP (U/L)	PTH (pg/mL)	Maternal Vitamin D	Pregnancy Vitamin D Supplementation	Infant Vitamin D Supplementation	Type of Feeding
Mallick et al., 2023 [19]	6 days	12	6.7	2.8	536	78.3	6.9	NM	No	BF
Kossof et al., 2002 [23]	34 days	<5	6.1	3	NM	129	Unknown	NM	NM	FF + BF
Levy-Shraga et al., 2015 [24]	2 months	4.5	6	3.36	1214	300	21	NM	400 IU/day	FF
Camadoo et al., 2007 [26]	7 days	14.8	6.33	NM	NM	64.1	Unknown	No	No	BF
Tran et al., 2022, [28]	3 months	4.3	7.56	2.52	NM	327.3	Unknown	NM	NM	BF
Arief et al., 2022 [33]	2 months	13.8	3.4	NM	373	235.9	Unknown	NM	No	BF
Bellazzini et al., 2005 [16]	8 months	1,25 Dihydroxy- vitamin D: 45 pg/mL	5.9	3.36	634	178	Unknown	NM	No	BF
Rodd et al., 2005 [35]	6 weeks	28	6.8	NM	1375	495	Unknown	NM	265 IU/day	Soy-based formula
	2 months	4.8	5	NM	686	32	Unknown	NM	No	MF (BF + Soy-based formula)
	2 months	Undetectable	5.2	3	Within normal range	220	Unknown	Yes, unknown amount	No	Soy-based formula
Vuletić et al., 2016 [36]	5 months	<3	5.08	1.44	878	283	Unknown	NM	No	FF for two months; afterwards cow milk and CF (started at 4 months of age)
Mantadakis et al., 2012 [37]	5 months	7.9	6.6	3.2	1079	245	Unknown	NM	NM	BF
Wallis et al., 2008 [38]	5 months	0.8	5.52	NM	970	NM	Unknown	No	NM	BF

Legend: +—and; ALP—alkaline phosphatase; BF—breastfeeding; CF—complementary feeding; FF—formula feeding; IU—international units; NM—not mentioned; PTH—parathormone.

## Data Availability

The original contributions presented in this study are included in the article. Further inquiries can be directed to the corresponding author.
